# Supplementary material for: Non-contact acoustic micro-tapping optical coherence elastography for quantification of corneal anisotropic elasticity: in vivo rabbit study
Source: ArXiv. 2023 Jan 25:arXiv:2301.10652v1. Preprint. [Version 1] (PMC9900963)
Supplement: 1 [file NIHPP2301.10652V1-supplement-1.pdf]

Supplemental Online Content

Non-contact acoustic micro-tapping optical coherence elastography for quantification of corneal anisotropic elasticity: in vivo rabbit study

Mitchell A. Kirby[1], Gabriel Regnault[1*], Ivan Pelivanov[1], Matthew O'Donnell[1], Ruikang K. Wang[1,3], Tueng T. Shen[2,3]

[1]*Department of Bioengineering, University of Washington, Seattle, Washington 98105, USA*
[2]*School of Medicine, University of Washington, Seattle, Washington 98195, USA*
[3]*Department of Ophthalmology, University of Washington, Seattle, Washington 98104, USA*
[*]*Correspondence: Gabriel Regnault, Department of Bioengineering, University of Washington, Seattle, Washington 98105, USA Email: gregnaul@uw.edu*

Supplemental Methods

1. Quality of fit and generation of uncertainty intervals

While g_{NITI} (Eq. 5, see Methods) provides an estimate for how well the theoretical A_0 mode matches experimental data, it alone does not provide confidence intervals on the output moduli G and μ. Due to the maximization approach for weighted fitting based on the energy in the 2-D Fourier transform use in this study, residual errors are not computed, making traditional confidence interval methods difficult to apply. For example, a low value of g_{NITI} would suggest that the A_0 dispersion curve calculated from the NITI model poorly described the actual dispersion measured within the sample. In such case, modulus estimates should have increased uncertainty.

To estimate uncertainty, G and μ were varied independently around the values near a maximum g_{NITI} and $\Phi(\mu, G)$ was recorded. For each combination of G and μ, Eq. 4 (Methods) was used to calculate $\Phi(\mu, G)$, which was then used to determine:

$$\psi(\mu, G) = \frac{\Phi(\mu,G)}{\Phi_{\max}} , \qquad (S.1)$$

where the ψ function represented goodness of fit values normalized to the maximum energy for a range of values. A representative example of $\psi(\mu, G)$ can be seen in Figure S1. Here (*x-t* plot shown in Figure S1a), the iterative routine converged on a best-fit A_0 mode (Figure S1b) where $g_{\mathrm{NITI}} = 0.98$ when $G = 20.5$ kPa and $\mu = 4$ MPa. In Figure S1c, ψ is shown for $G \in$ [10 – 40] kPa and $\mu \in$ [1 – 50] MPa. It highlights the high sensitivity of ψ to a change in G while it remains relatively stable while μ varies. In Figure S1d, ψ is shown when $\mu = 4$ MPa and G was varied from 10 kPa- 40 kPa. The corresponding A_0 dispersion curves (constitutive equation found in Ref[2]) as G varied can be seen in Figure S1f. As described previously, the high-frequency threshold of the A_0 dispersion curve is largely determined by G. In Figure S1e, ψ is shown when $G = 20.5$ kPa and μ swept across a 1 MPa - 50 MPa range. Again, the corresponding A_0 dispersion curves can be seen in Figure S1g. As suggested previously, the A_0 mode is not as sensitive to changes in μ for the degree of shear anisotropy (μ/G) expected in the cornea. As such, ψ is less sensitive to changes in μ and produces higher relative uncertainty (see also Figure S1c). This routine provided a range for both G and μ values indicating the degree to which the iterative solution converged on a single value.

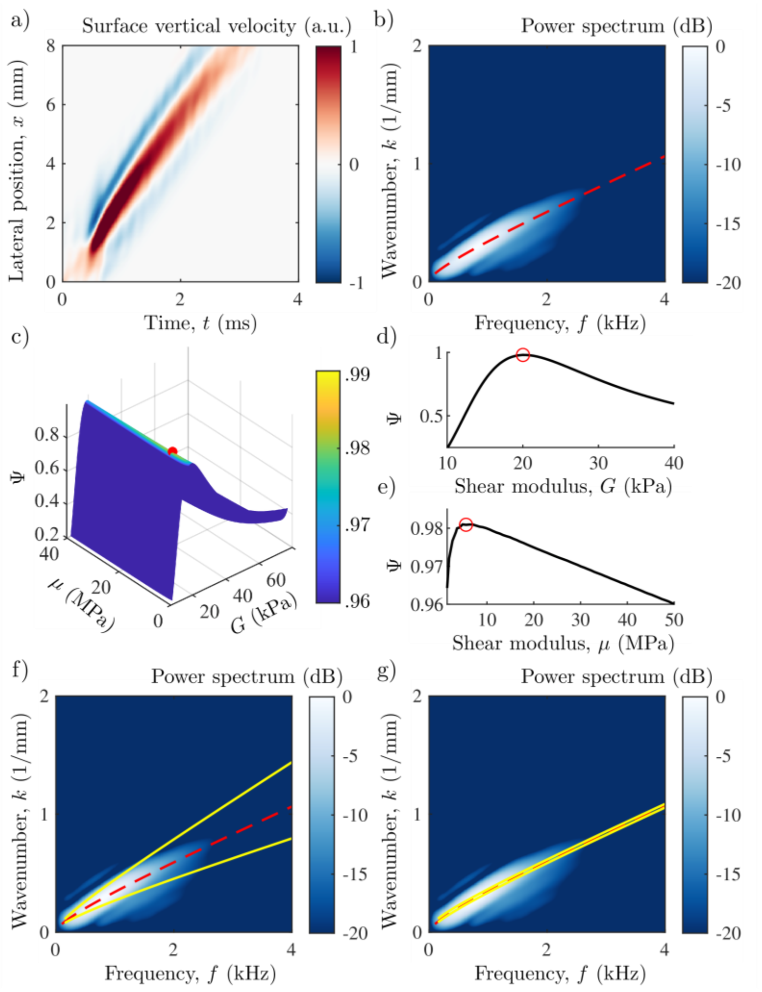

Figure S1. a) Space-time (*x*-*t*) plot of surface vibrations measured via OCT in a normal cornea sample. b) Best-fit solution to the dispersion equation in *k*-*f* space (based on a unique combination of elastic moduli, μ and G, displayed in red) on top of experimentally obtained A_0 mode for the corresponding cornea; c) Surface plot of ψ for $G \in$ [10 - 40] kPa and $\mu \in$ [1 - 50] MPa. d) ψ for $\mu = 4$ MPa, where G was swept across a range from 10 kPa to 40 kPa. The red dot shows the highest value of ψ e) ψ for $G = 20.5$ kPa, where μ was swept across a range of 1 MPa - 50 MPa. Yellow lines plotted on top of measured energy in the *k*-*f* domain are A_0 dispersion curves corresponding to the range

f) G = 10 kPa and G = 40 kPa, with $\mu = 4$ MPa and g) μ= 1 MPa and μ= 50 MPa, with $G = 20.5$ kPa.

Due to the shape of ψ, the uncertainty in the fit produced uneven error bars. Note that the absolute value of g_{NITI} provides an estimate for model error, where $g_{\mathrm{NITI}} = 1$ for a NITI material would have very small uncertainty intervals. As g_{NITI} is reduced, the shape of ψ widens for both moduli and model uncertainty increases.

Because n=5 repeat scans were taken, five independent measurements produced corresponding values for G and μ and their respective uncertainty ranges. The uncertainty ranges for each cornea (at each IOP) were finally calculated by taking the square-root of the means of the upper and lower limits, divided by the number of scans (n=5).

2. Exclusion criteria

In cases where the NITI model does not describe measured wavefields properly (due to poor excitation, misalignment, corneal structure abnormalities, etc.), the iterative fitting routine will arrive at a modulus value that has little to no physical meaning. To determine cases where the measured wavefields are accurately described by the NITI model, a 'cut-off' criterion in the goodness of fit for both G and μ was determined. Any scan with a goodness of fit below the cut-off value was omitted from analysis.

To determine the relationship between g_{NITI} and model error in rabbit corneas, an iterative fit was performed and g_{NITI} calculated for all OCE scans (at all pressures), providing g_{NITI}/moduli histograms (Figures S2a-b). Each section of the histograms corresponds to

a 0.01 range in g_{NITI}, 5 kPa for G, and 2 MPa for μ. These histograms illustrate the repartition of moduli obtained from the fits and show, as expected, a stable repartition of moduli in the high goodness of fit range.

In order to determine critical g_{NITI} values, histograms were integrated in the g_{NITI} direction. For every modulus range, the number of occurrences was counted and the mean g_{NITI} computed. These results are shown in Figures S2c-d. The modulus clearly increases (associated with faster wave speeds) as g_{NITI} decreases progressively before approaching a point where this behavior breaks, reaching a plateau. The plateau is associated with nearly random statistics for reconstructed moduli, corresponding to the range of g_{NITI} below which the fitting procedure used for moduli reconstruction is inaccurate. The final cutoff value was computed as the average g_{NITI} over the plateau range. Table S1 presents the cutoff goodness of fit (g_{NITI}) criteria determined by the method detailed above for both G and μ

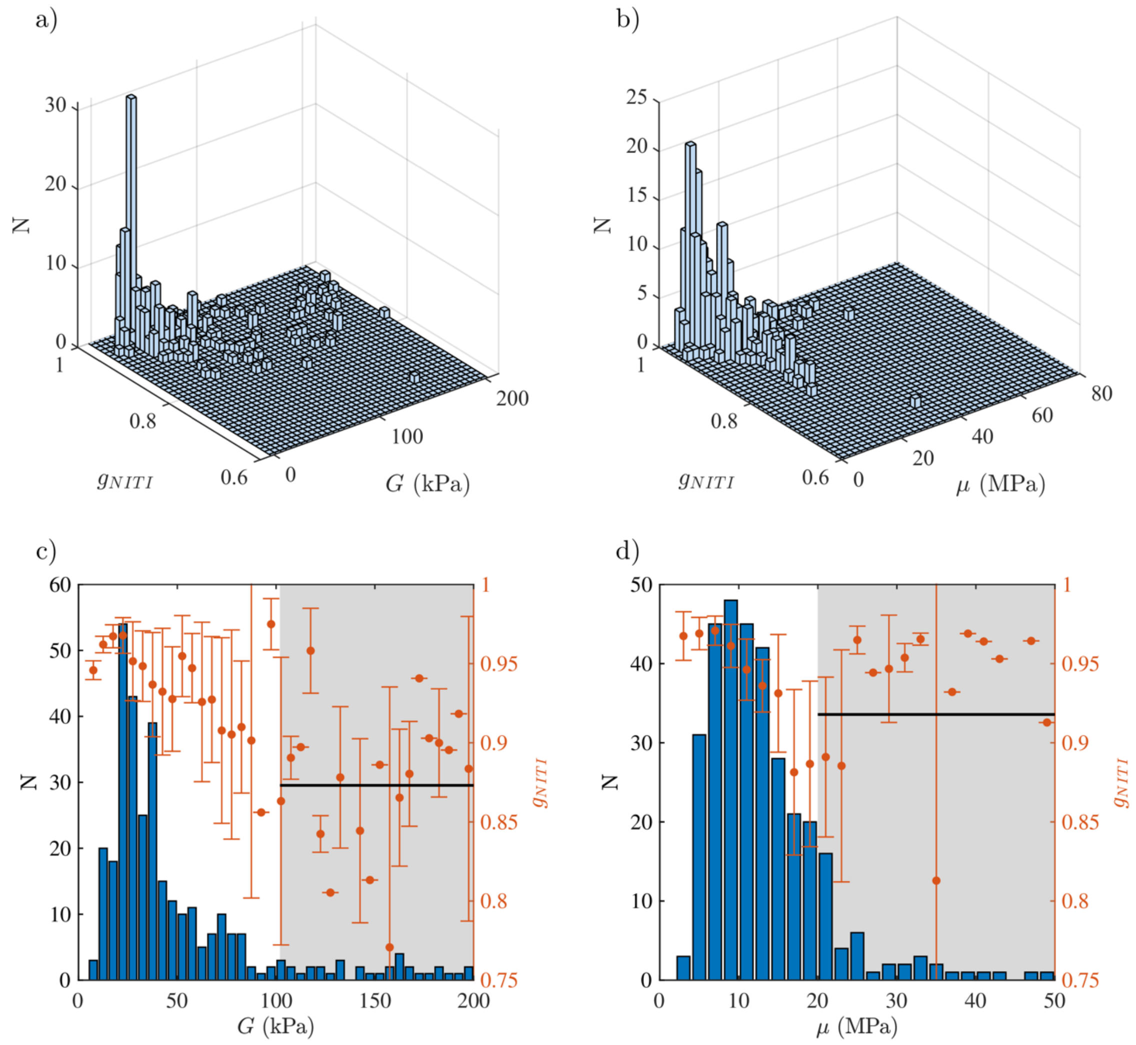

Figure S2. Procedure to determine the criteria of exclusion for G and μ. a), b) 2D histograms illustrating the distribution of fitted moduli, respectively for G and μ, and the goodness of fit, g_{NITI}, metric. c), d) 1D histogram illustrating the distribution of data as a function of the fitted moduli, respectively for G and μ. The right axis represents the averaged g_{NITI} in the considered modulus range and the error bars represent the standard deviation in the measured goodness of fit. The black line and gray shaded area indicate the range over which the cutoff g_{NITI} was calculated. Exact values for the cutoff g_{NITI} are given in Table S1).

Table S1: Cut off values of goodness of fit, g_{NITI}

Modulus	Cutoff value (g_{NITI})
G	0.87
μ	0.92

3. Mechanical testing results for individual cornea samples

Each cornea was first tested with OCE in vivo, where at least 10 non-contact OCE scans were performed on each eye. Once in vivo measurements were performed, the pressure was measured with a Tono-Pen. Following in vivo measurements, euthanasia was performed and whole globe corneas were harvested. Whole globes were placed in a mold containing a damp sterile cotton pad to stabilize samples and mimic in vivo boundary conditions. A 20-gauge needle connected to a bath filled with BSS was inserted through the temporal wall of the sclera to apply a controlled internal hydrostatic pressure (IOP) ranging from 1 mmHg to 23 mmHg. Each sample was scanned at room temperature and imaging took no longer than 1 hour per sample. After AμT-OCE, corneal buttons were cut and rheometry measurements were performed. Each corneal button then was sectioned into strips approximately 6 mm wide along the nasotemporal axis of the cornea and subject to tensile testing up to 10% strain. The elastic moduli quantified from all tests, for all corneas, are shown below (Figure S3- Figure S11). All ex vivo data were acquired within 6 hours of animal euthanasia. The corresponding g_{NITI} for all OCE measurements are also included in Figure S12- Figure S20.

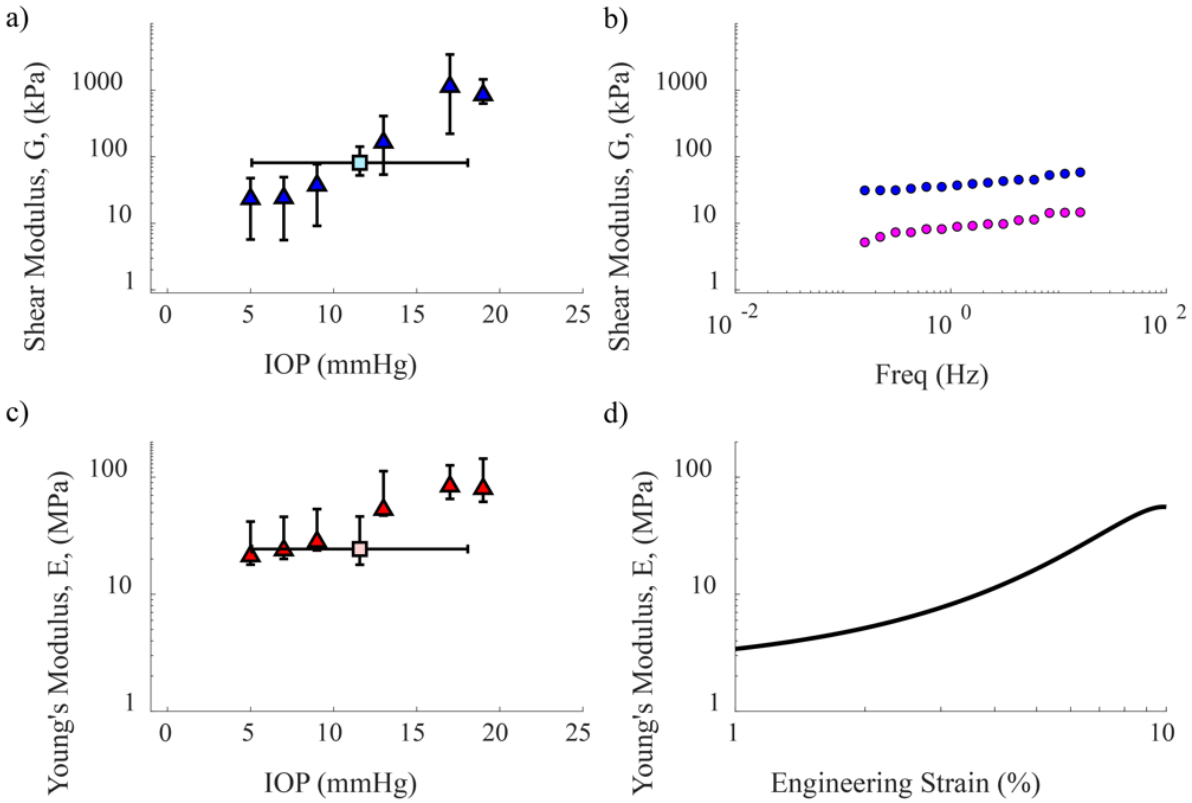

Figure S3. Measurement of anisotropic elastic moduli in Cornea #1. a) Out-of-plane shear modulus, G, measured with AμT-OCE in vivo (square) and ex vivo at controlled pressure (triangle). The vertical error bars correspond with uncertainty intervals and the horizontal error bar corresponds with in vivo IOP uncertainty. b) Out-of-plane shear modulus, G, measured with parallel plate rheometry. Blue corresponds with storage modulus and pink with loss modulus. c) In-plane Young's modulus, E, measured with AμT-OCE in vivo (square) and ex vivo at controlled pressure (triangle). The vertical error bars correspond with uncertainty intervals and the horizontal error bar corresponds with in vivo IOP uncertainty. d) Strain-dependent Young's moduli, E, measured via extension testing up to 10% strain, or where visible tissue damage occurred.

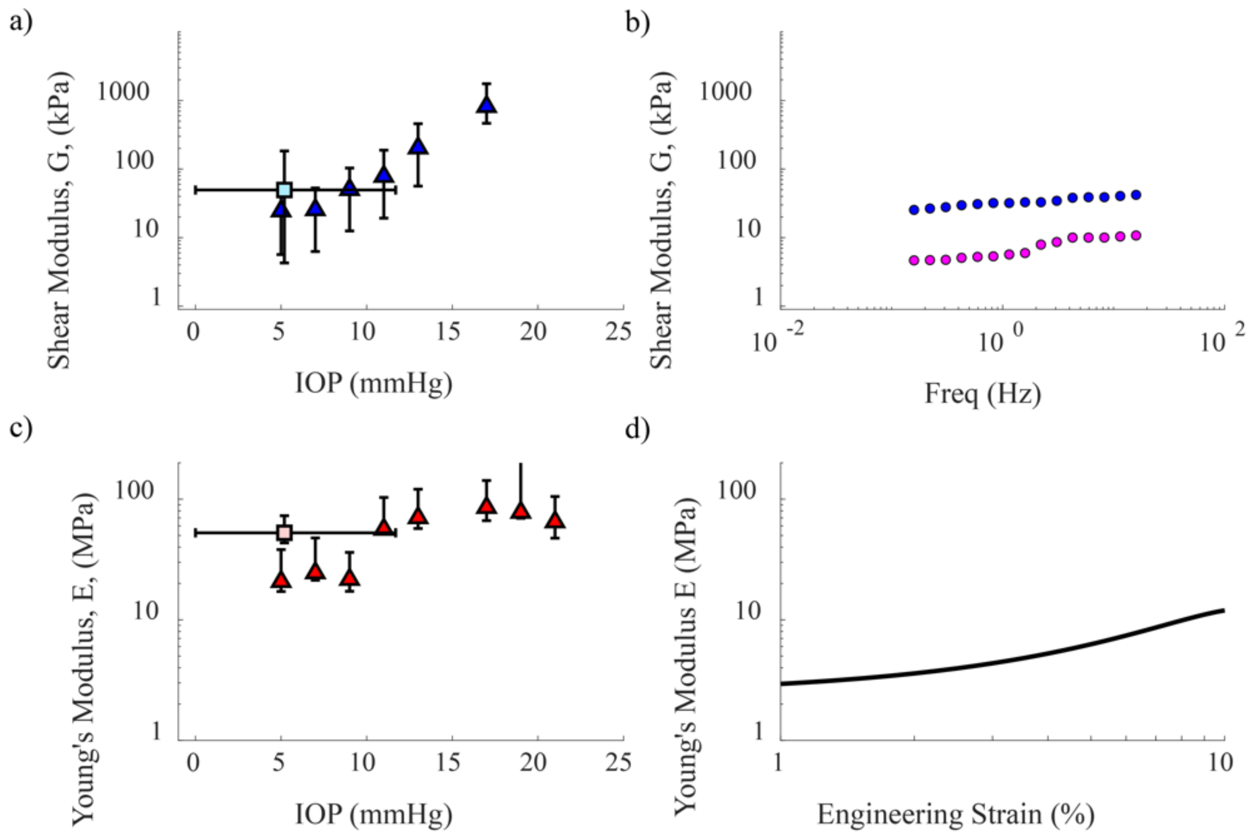

Figure S4. Measurement of anisotropic elastic moduli in Cornea #2. a) Out-of-plane shear modulus, G, measured with AμT-OCE in vivo (square) and ex vivo at controlled pressure (triangle). The vertical error bars correspond with uncertainty intervals and the horizontal error bar corresponds with in vivo IOP uncertainty. b) Out-of-plane shear modulus, G, measured with parallel plate rheometry. Blue corresponds with storage modulus and pink with loss modulus. c) In-plane Young's modulus, E, measured with AμT-OCE in vivo (square) and ex vivo at controlled pressure (triangle). The vertical error bars correspond with uncertainty intervals and the horizontal error bar corresponds with in vivo IOP uncertainty. d) Strain-dependent Young's moduli, E, measured via extension testing up to 10% strain, or where visible tissue damage occurred.

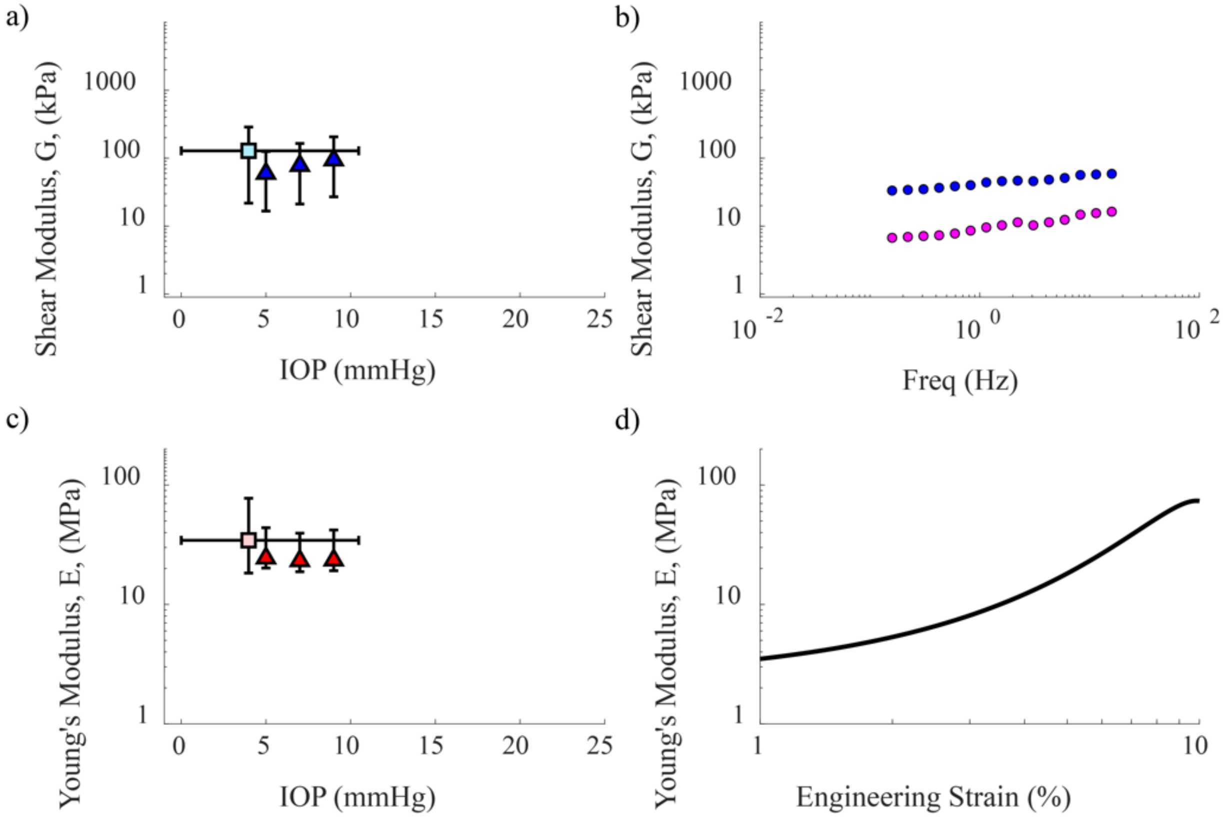

Figure S5. Measurement of anisotropic elastic moduli in Cornea #3. a) Out-of-plane shear modulus, G, measured with AμT-OCE in vivo (square) and ex vivo at controlled pressure (triangle). The vertical error bars correspond with uncertainty intervals and the horizontal error bar corresponds with in vivo IOP uncertainty. b) Out-of-plane shear modulus, G, measured with parallel plate rheometry. Blue corresponds with storage modulus and pink with loss modulus. c) In-plane Young's modulus, E, measured with AμT-OCE in vivo (square) and ex vivo at controlled pressure (triangle). The vertical error bars correspond with uncertainty intervals and the horizontal error bar corresponds with in vivo IOP uncertainty. d) Strain-dependent Young's moduli, E, measured via extension testing up to 10% strain, or where visible tissue damage occurred.

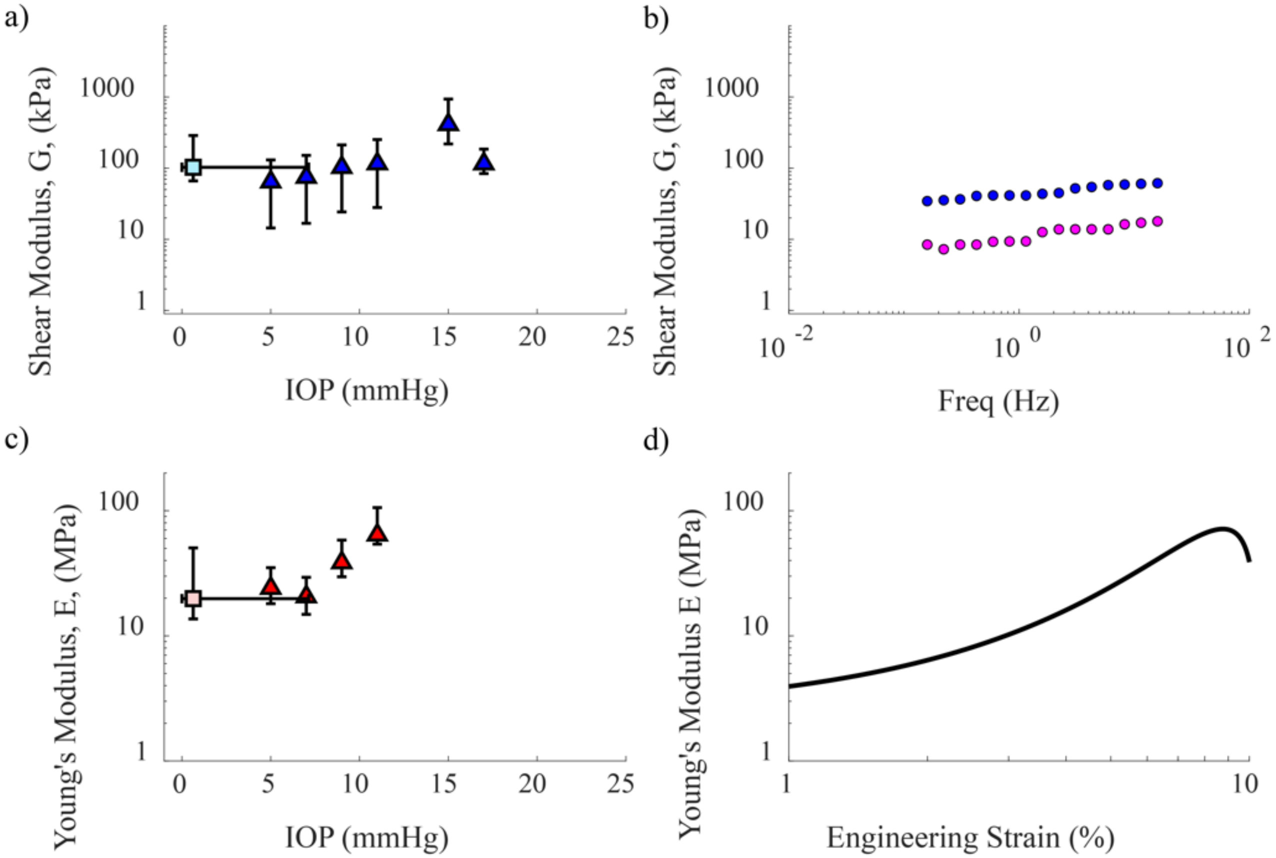

Figure S6. Measurement of anisotropic elastic moduli in Cornea #4. a) Out-of-plane shear modulus, G, measured with AμT-OCE in vivo (square) and ex vivo at controlled pressure (triangle). The vertical error bars correspond with uncertainty intervals and the horizontal error bar corresponds with in vivo IOP uncertainty. b) Out-of-plane shear modulus, G, measured with parallel plate rheometry. Blue corresponds with storage modulus and pink with loss modulus. c) In-plane Young's modulus, E, measured with AμT-OCE in vivo (square) and ex vivo at controlled pressure (triangle). The vertical error bars correspond with uncertainty intervals and the horizontal error bar corresponds with in vivo IOP uncertainty. d) Strain-dependent Young's moduli, E, measured via extension testing up to 10% strain, or where visible tissue damage occurred.

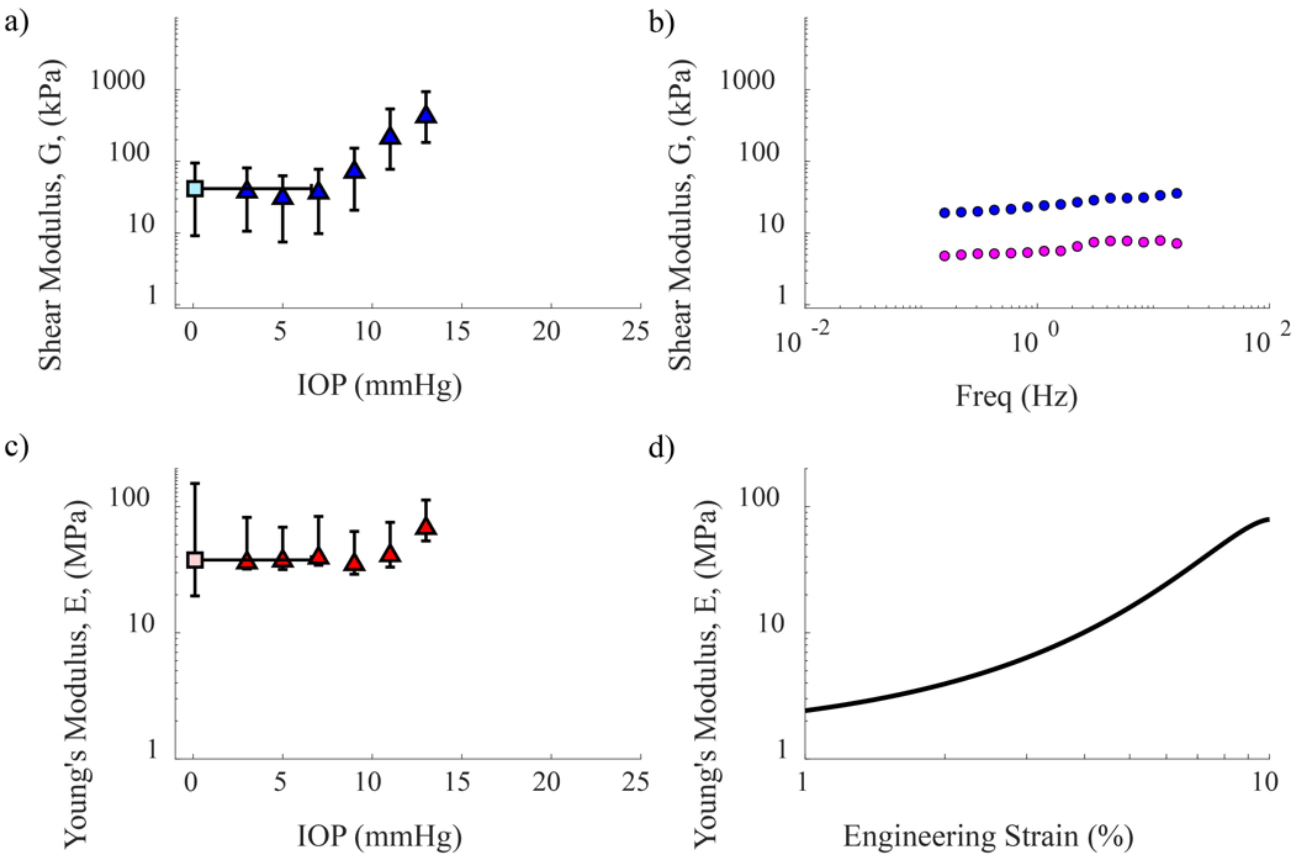

Figure S7. Measurement of anisotropic elastic moduli in Cornea #5. a) Out-of-plane shear modulus, G, measured with AμT-OCE in vivo (square) and ex vivo at controlled pressure (triangle). The vertical error bars correspond with uncertainty intervals and the horizontal error bar corresponds with in vivo IOP uncertainty. b) Out-of-plane shear modulus, G, measured with parallel plate rheometry. Blue corresponds with storage modulus and pink with loss modulus. c) In-plane Young's modulus, E, measured with AμT-OCE in vivo (square) and ex vivo at controlled pressure (triangle). The vertical error bars correspond with uncertainty intervals and the horizontal error bar corresponds with in vivo IOP uncertainty. d) Strain-dependent Young's moduli, E, measured via extension testing up to 10% strain, or where visible tissue damage occurred.

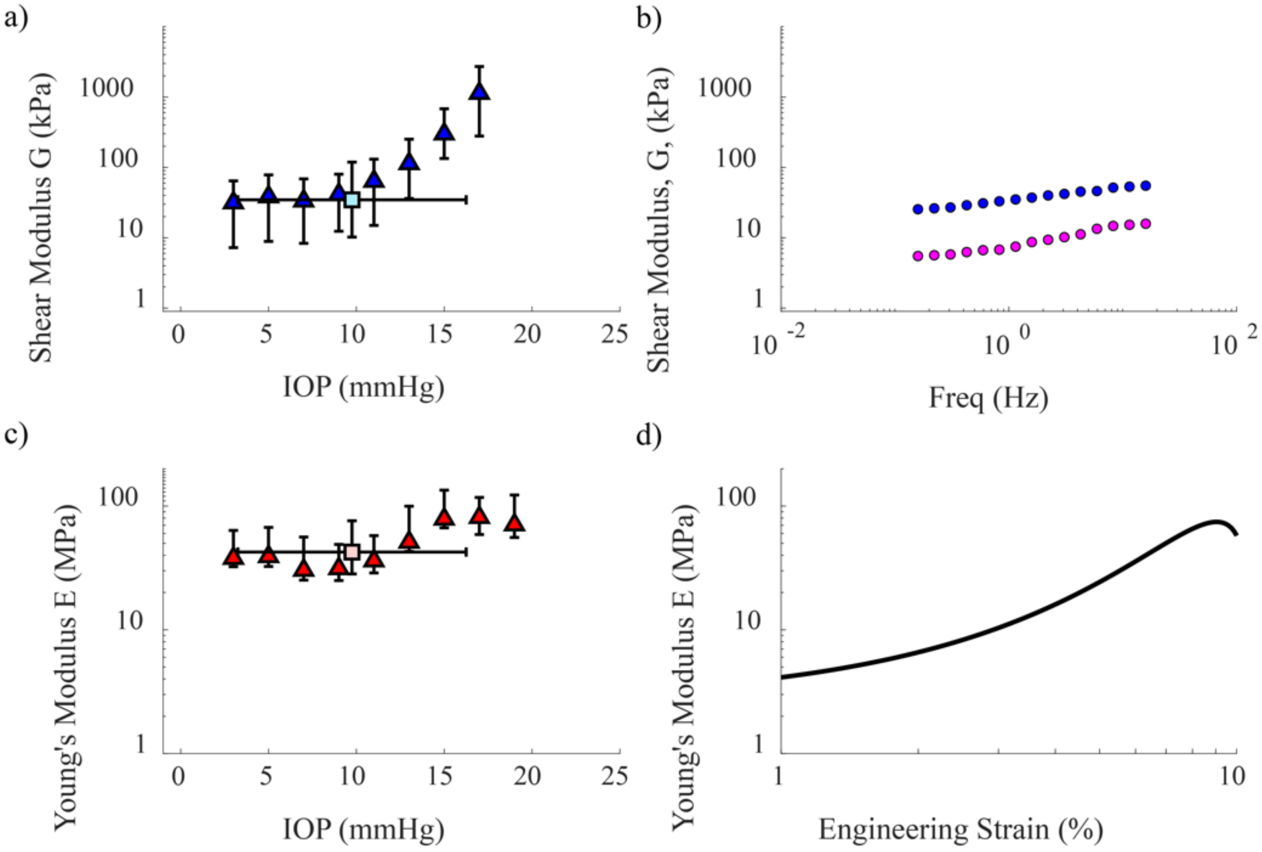

Figure S8. Measurement of anisotropic elastic moduli in Cornea #6. a) Out-of-plane shear modulus, G, measured with AμT-OCE in vivo (square) and ex vivo at controlled pressure (triangle). The vertical error bars correspond with uncertainty intervals and the horizontal error bar corresponds with in vivo IOP uncertainty. b) Out-of-plane shear modulus, G, measured with parallel plate rheometry. Blue corresponds with storage modulus and pink with loss modulus. c) In-plane Young's modulus, E, measured with AμT-OCE in vivo (square) and ex vivo at controlled pressure (triangle). The vertical error bars correspond with uncertainty intervals and the horizontal error bar corresponds with in vivo IOP uncertainty. d) Strain-dependent Young's moduli, E, measured via extension testing up to 10% strain, or where visible tissue damage occurred.

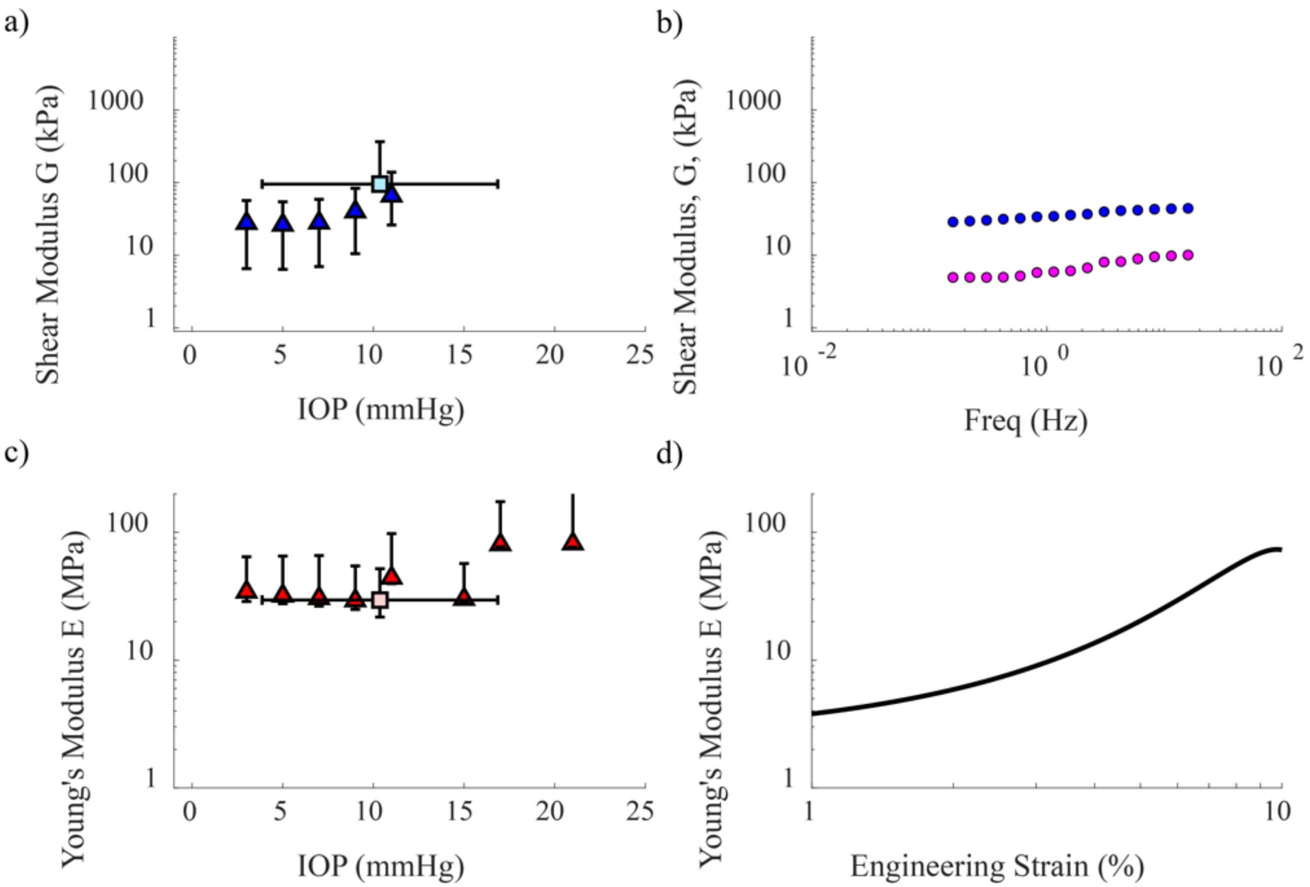

Figure S9. Measurement of anisotropic elastic moduli in Cornea #7. a) Out-of-plane shear modulus, G, measured with AμT-OCE in vivo (square) and ex vivo at controlled pressure (triangle). The vertical error bars correspond with uncertainty intervals and the horizontal error bar corresponds with in vivo IOP uncertainty. b) Out-of-plane shear modulus, G, measured with parallel plate rheometry. Blue corresponds with storage modulus and pink with loss modulus. c) In-plane Young's modulus, E, measured with AμT-OCE in vivo (square) and ex vivo at controlled pressure (triangle). The vertical error bars correspond with uncertainty intervals and the horizontal error bar corresponds with in vivo IOP uncertainty. d) Strain-dependent Young's moduli, E, measured via extension testing up to 10% strain, or where visible tissue damage occurred.

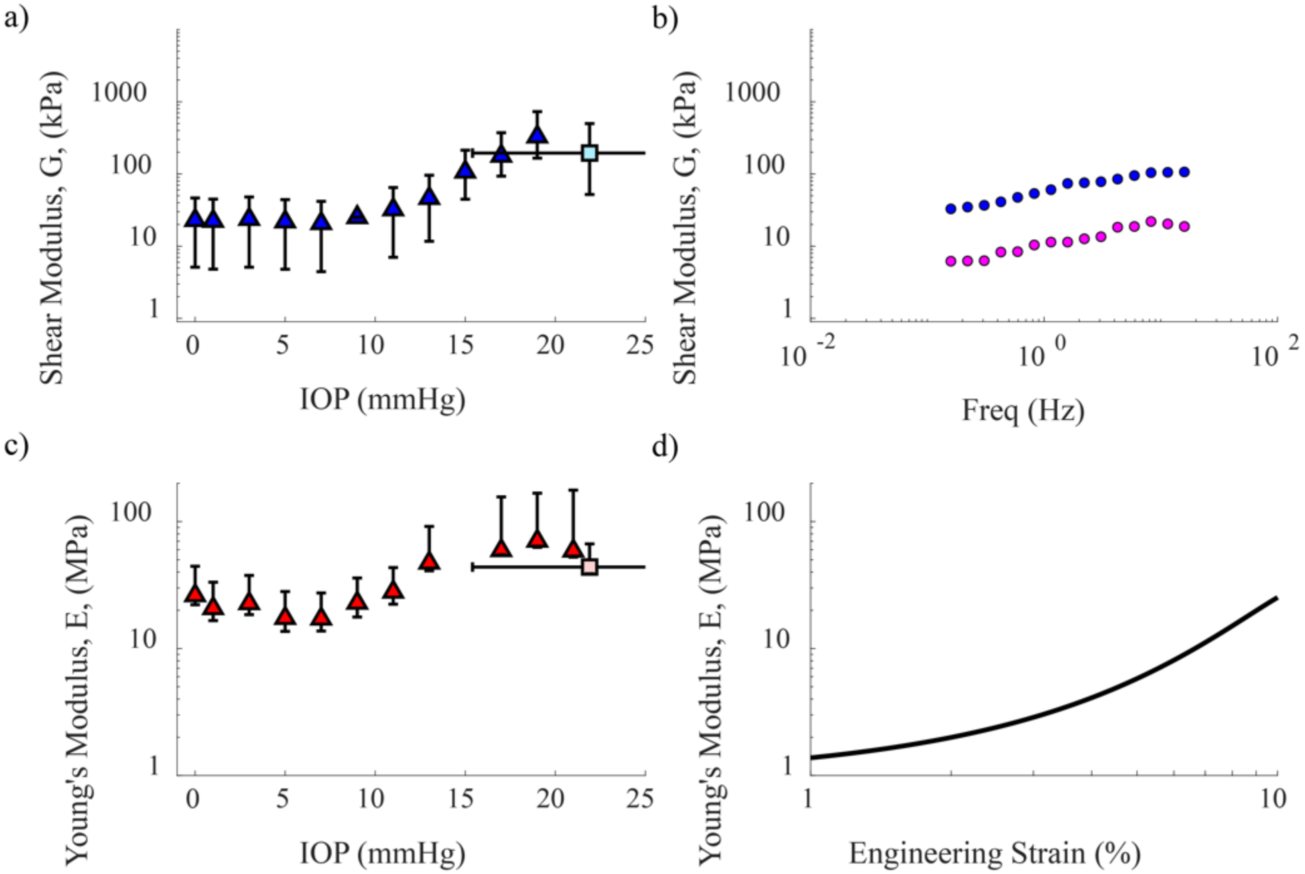

Figure S10. Measurement of anisotropic elastic moduli in Cornea #8. a) Out-of-plane shear modulus, G, measured with AμT-OCE in vivo (square) and ex vivo at controlled pressure (triangle). The vertical error bars correspond with uncertainty intervals and the horizontal error bar corresponds with in vivo IOP uncertainty. b) Out-of-plane shear modulus, G, measured with parallel plate rheometry. Blue corresponds with storage modulus and pink with loss modulus. c) In-plane Young's modulus, E, measured with AμT-OCE in vivo (square) and ex vivo at controlled pressure (triangle). The vertical error bars correspond with uncertainty intervals and the horizontal error bar corresponds with in vivo IOP uncertainty. d) Strain-dependent Young's moduli, E, measured via extension testing up to 10% strain, or where visible tissue damage occurred.

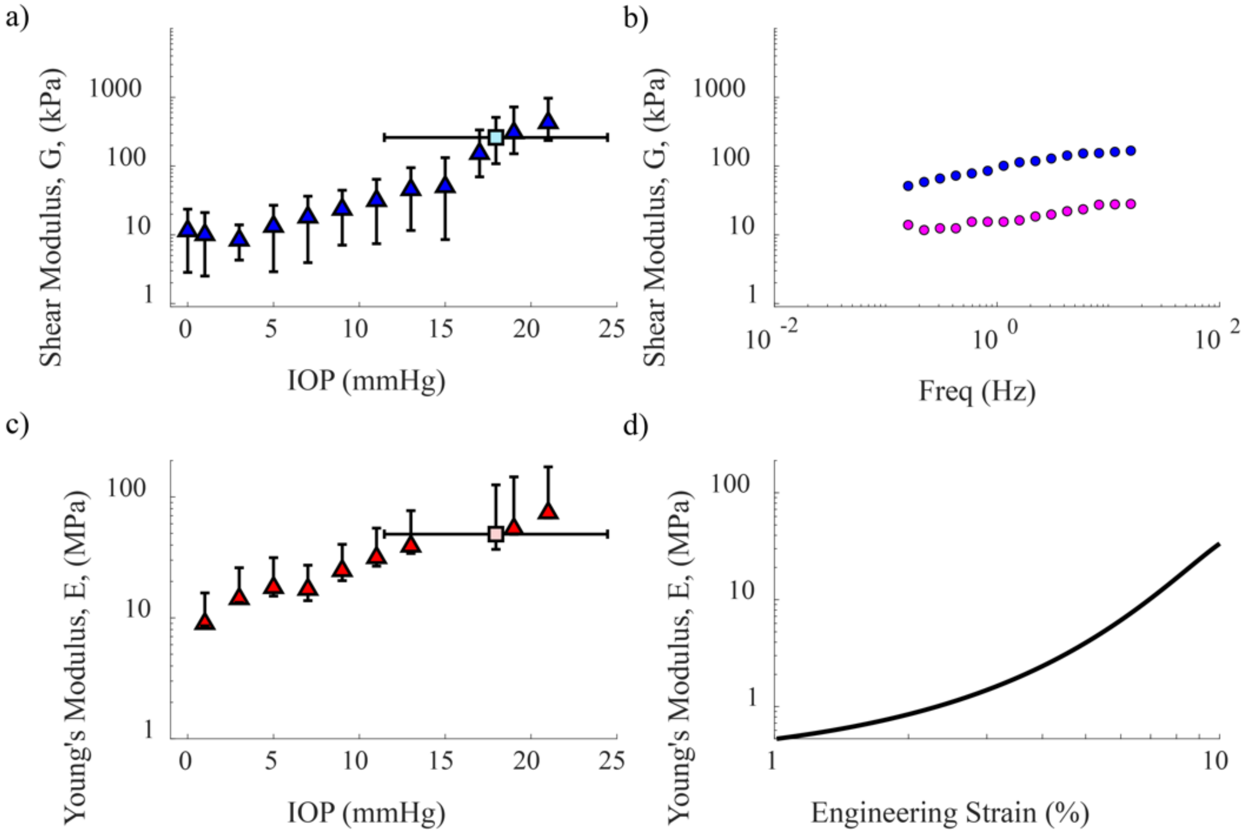

Figure S11. Measurement of anisotropic elastic moduli in Cornea #9. a) Out-of-plane shear modulus, G, measured with AμT-OCE in vivo (square) and ex vivo at controlled pressure (triangle). The vertical error bars correspond with uncertainty intervals and the horizontal error bar corresponds with in vivo IOP uncertainty. b) Out-of-plane shear modulus, G, measured with parallel plate rheometry. Blue corresponds with storage modulus and pink with loss modulus. c) In-plane Young's modulus, E, measured with AμT-OCE in vivo (square) and ex vivo at controlled pressure (triangle). The vertical error bars correspond with uncertainty intervals and the horizontal error bar corresponds with in vivo IOP uncertainty. d) Strain-dependent Young's moduli, E, measured via extension testing up to 10% strain, or where visible tissue damage occurred.

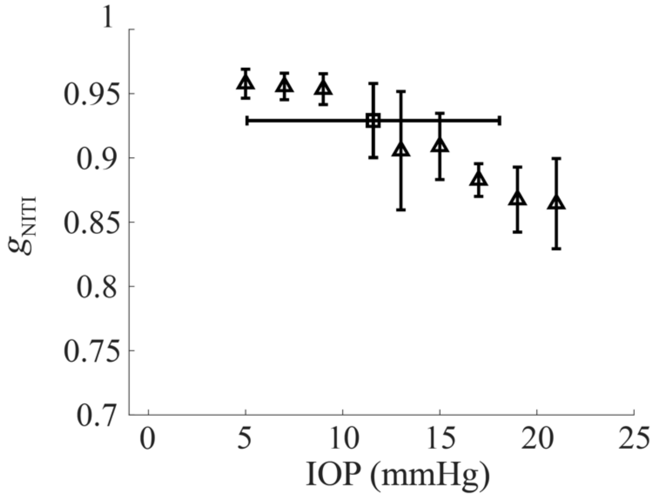

Figure S12. Mean goodness of fit (g_{NITI}) for all OCE scans in Cornea #1. The triangles correspond with ex vivo measurements at controlled IOP and error bars correspond with standard deviation across 5 repeat scans. The square corresponds with in vivo measurements and vertical error bars associate with the standard deviation of g_{NITI} across at least 5 repeat scans. Horizontal error bar corresponds with in vivo IOP uncertainty.

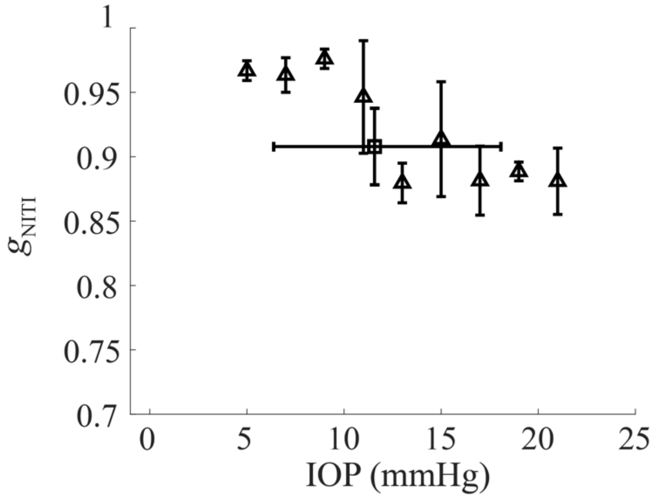

Figure S13. Mean goodness of fit (g_{NITI}) for all OCE scans in Cornea #2. The triangles correspond with ex vivo measurements at controlled IOP and error bars correspond with

standard deviation across 5 repeat scans. The square corresponds with in vivo measurements and vertical error bars associate with the standard deviation of g_{NITI} across at least 5 repeat scans. Horizontal error bar corresponds with in vivo IOP uncertainty.

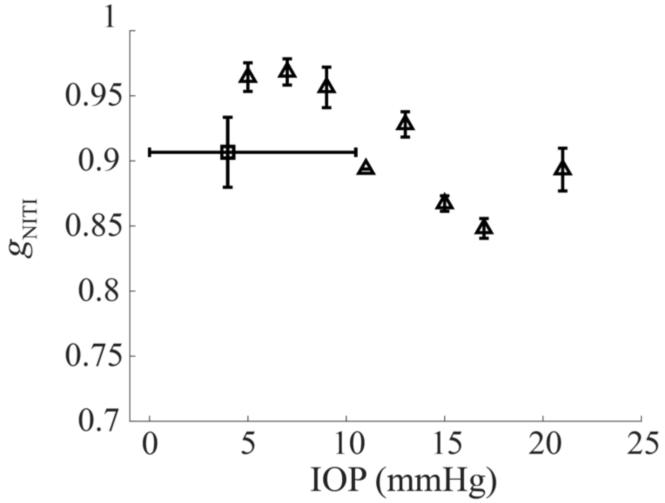

Figure S14. Mean goodness of fit (g_{NITI}) for all OCE scans in Cornea #3. The triangles correspond with ex vivo measurements at controlled IOP and error bars correspond with standard deviation across 5 repeat scans. The square corresponds with in vivo measurements and vertical error bars associate with the standard deviation of g_{NITI} across at least 5 repeat scans. Horizontal error bar corresponds with in vivo IOP uncertainty.

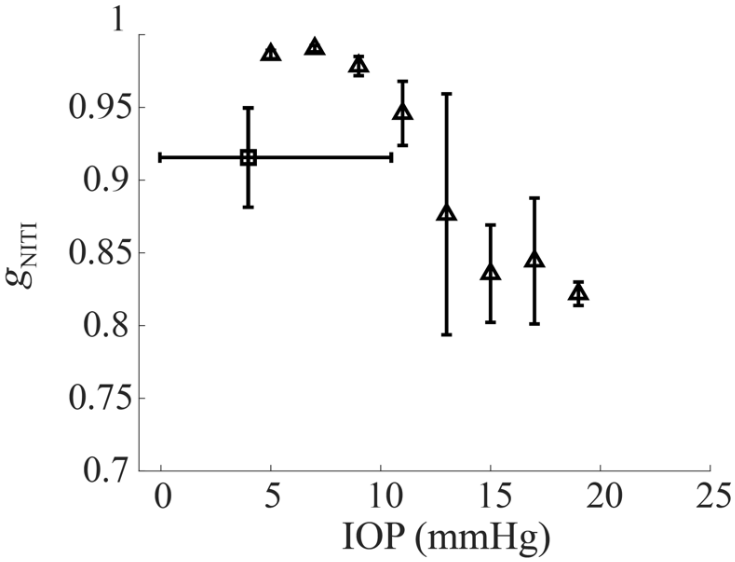

Figure S15. Mean goodness of fit (g_{NITI}) for all OCE scans in Cornea #4. The triangles correspond with ex vivo measurements at controlled IOP and error bars correspond with standard deviation across 5 repeat scans. The square corresponds with in vivo measurements and vertical error bars associate with the standard deviation of g_{NITI} across at least 5 repeat scans. Horizontal error bar corresponds with in vivo IOP uncertainty.

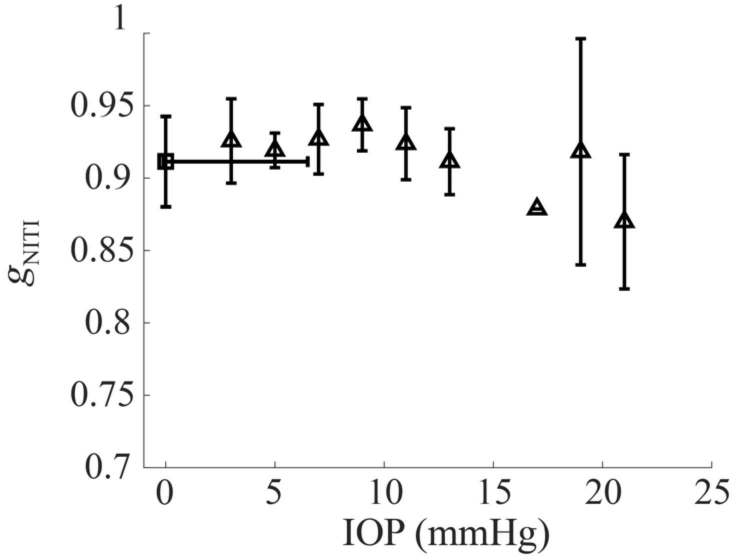

Figure S16. Mean goodness of fit (g_{NITI}) for all OCE scans in Cornea #5. The triangles correspond with ex vivo measurements at controlled IOP and error bars correspond with standard deviation across 5 repeat scans. The square corresponds with in vivo measurements and vertical error bars associate with the standard deviation of g_{NITI} across at least 5 repeat scans. Horizontal error bar corresponds with in vivo IOP uncertainty.

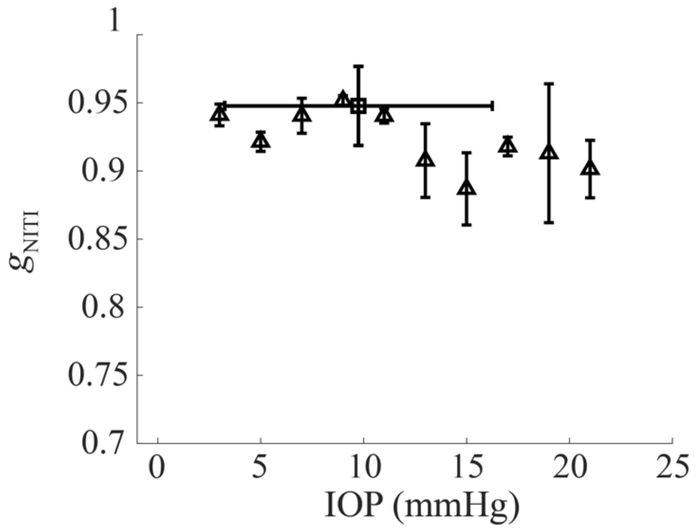

Figure S17. Mean goodness of fit (g_{NITI}) for all OCE scans in Cornea #6. The triangles correspond with ex vivo measurements at controlled IOP and error bars correspond with standard deviation across 5 repeat scans. The square corresponds with in vivo measurements and vertical error bars associate with the standard deviation of g_{NITI} across at least 5 repeat scans. Horizontal error bar corresponds with in vivo IOP uncertainty.

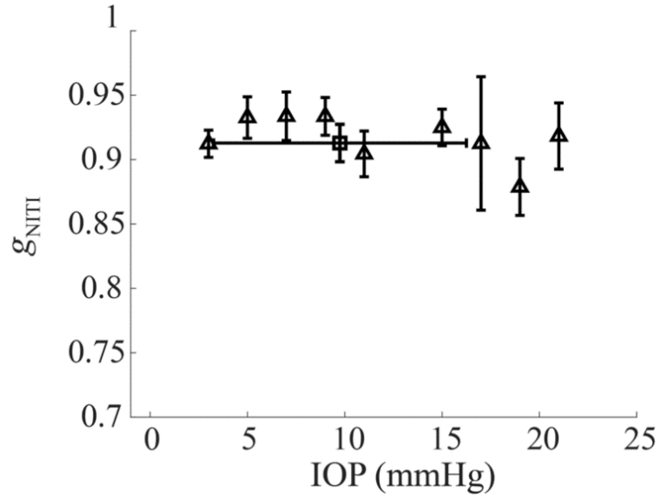

Figure S18. Mean goodness of fit (g_{NITI}) for all OCE scans in Cornea #7. The triangles correspond with ex vivo measurements at controlled IOP and error bars correspond with standard deviation across 5 repeat scans. The square corresponds with in vivo measurements and vertical error bars associate with the standard deviation of g_{NITI} across at least 5 repeat scans. Horizontal error bar corresponds with in vivo IOP uncertainty.

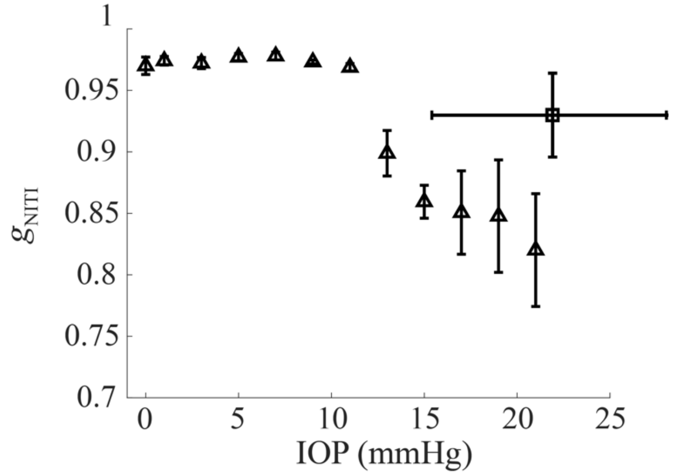

Figure S19. Mean goodness of fit (g_{NITI}) for all OCE scans in Cornea #8. The triangles correspond with ex vivo measurements at controlled IOP and error bars correspond with

standard deviation across 5 repeat scans. The square corresponds with in vivo measurements and vertical error bars associate with the standard deviation of g_{NITI} across at least 5 repeat scans. Horizontal error bar corresponds with in vivo IOP uncertainty.

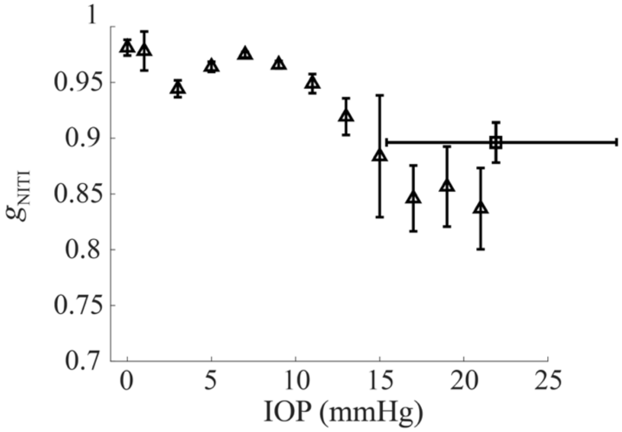

Figure S20. Mean goodness of fit (g_{NITI}) for all OCE scans in Cornea #9. The triangles correspond with ex vivo measurements at controlled IOP and error bars correspond with standard deviation across 5 repeat scans. The square corresponds with in vivo measurements and vertical error bars associate with the standard deviation of g_{NITI} across at least 5 repeat scans. Horizontal error bar corresponds with in vivo IOP uncertainty.

Supplemental References

1. Pitre, J. J. *et al.* Nearly-incompressible transverse isotropy (NITI) of cornea elasticity: model and experiments with acoustic micro-tapping OCE. *Sci. Rep.* **10**, 1–14 (2020).
2. Kirby, M. A. *et al.* Delineating corneal elastic anisotropy in a porcine model using non-contact optical coherence elastography and ex vivo mechanical tests. *Ophthalmol. Sci.* 100058 (2021). doi:https://doi.org/10.1016/j.xops.2021.100058
